# Proteomic analysis of human lacrimal and tear fluid in dry eye disease

**DOI:** 10.1038/s41598-017-13817-y

**Published:** 2017-10-17

**Authors:** Jae Hun Jung, Yong Woo Ji, Ho Sik Hwang, Jae Won Oh, Hyun Chang Kim, Hyung Keun Lee, Kwang Pyo Kim

**Affiliations:** 1 Department of Applied Chemistry, College of Applied Science, Kyung Hee University, Yongin, Korea; 2 Institute of Vision Research, Department of Ophthalmology, Yonsei University College of Medicine, Seoul, Korea; 3 Department of Ophthalmology, Chuncheon Sacred Heart Hospital, Hallym University, Chuncheon, Korea; 4 Department of Preventive Medicine, Yonsei University College of Medicine, Seoul, Korea

## Abstract

To understand the pathophysiology of dry eye disease (DED), it is necessary to characterize proteins in the ocular surface fluids, including tear fluid (TF) and lacrimal fluid (LF). There have been several reports of TF proteomes, but few proteomic studies have examined LF secreted from the lacrimal gland (LG). Therefore, we characterized the proteins constituting TF and LF by liquid chromatography mass spectrometry. TF and LF were collected from patients with non-Sjögren syndrome DED and from healthy subjects. Through protein profiling and label-free quantification, 1165 proteins from TF and 1448 from LF were identified. In total, 849 proteins were present in both TF and LF. Next, candidate biomarkers were verified using the multiple reaction monitoring assay in both TF and LF of 17 DED patients and 17 healthy controls. As a result, 16 marker proteins were identified (fold-change > 1.5, *p*-value < 0.05), of which 3 were upregulated in TF and 8 up- and 5 down-regulated in LF. In conclusion, this study revealed novel DED markers originating from the LG and tears by in-depth proteomic analysis and comparison of TF and LF proteins.

## Introduction

Analysis of human body fluids is important for identifying the cause of disease and biomarkers for diagnosing the disease^1,2^. The numerous and heterogeneous components carried by these fluids represent a rich source of information for therapeutic interventions. Among the sources for liquid biopsy, tear fluid (TF) representing ocular fluid is not frequently used, but is a good source for non-invasive investigation, as it is easy to assess and clear and it exhibits limited bacterial contamination. Therefore, TF has been used to diagnose various ocular and systemic diseases such as glaucoma^3,4^, dry eye disease (DED)^1,5,6^, thyroid-associated orbitopathy^7^, diabetes mellitus^8^, and cancer^9–11^.

DED is one of the most ubiquitous ailments confronting ophthalmologists. Schein *et al*. found that nearly 15% of people aged over 65 years had DED^12,13^. However, reliable epidemiological data regarding DED is limited by the lack of agreement regarding precise clinical definitions and reliable diagnostic tests for confirming the diagnosis^14,15^. Therefore, identifying reliable biomarkers for diagnosis, assessment of disease severity, and evaluation of prognosis is essential for understanding the pathophysiology of the disease and treating DED patients.

Previously, several studies have outlined the proteomic analysis of TF from patients with DED^16–19^. Although most TF is produced in the lacrimal gland (LG)^20^, ocular surface epithelial cells^21^, stromal immune cells^22^, and meibomian gland acinar cells also contribute components to the TF. However, lacrimal fluid (LF), which is distinct from TF, is an aqueous fluid secreted directly and solely from the LG. Therefore, it is necessary to understand the differences in protein composition between TF and LF to better evaluate the pathological status of the ocular surface and other parts of the body. However, few studies have examined and compared LG fluid proteomics with TF proteomics.

The purpose of this study was to profile the proteome of human LF and TF by high-resolution accurate-mass mass spectrometry (MS) and a targeted multiple reaction monitoring (MRM) assay. Furthermore, we investigated disease-specific biomarkers in prospective manner by comparing individual proteomes in TF and LF between normal and DED groups.

## Results

### Comprehensive global proteome profiling of TF and LF

**Figure 1:**
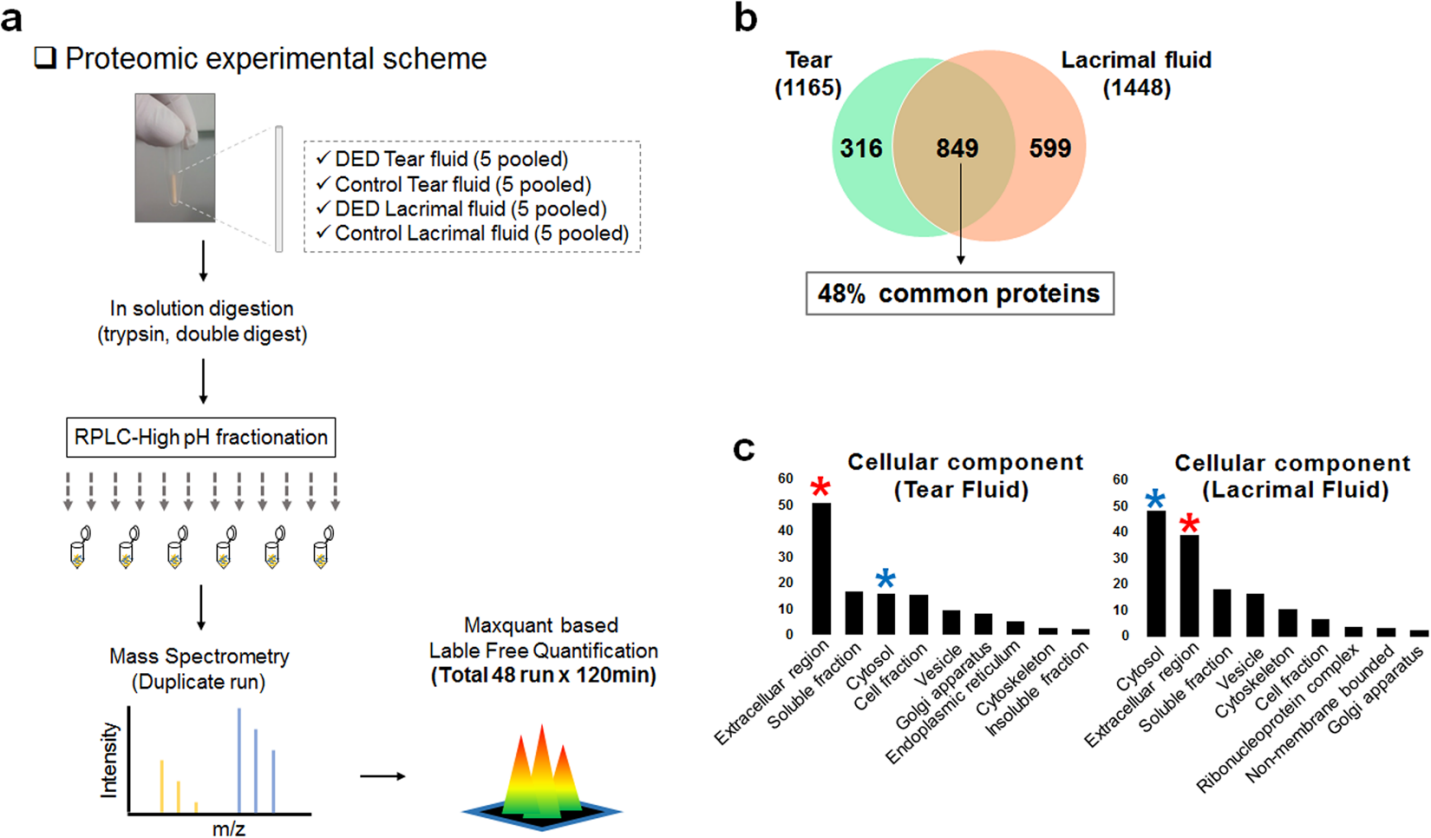
(**a**) Schematic diagram of proteomics experiments. Tear fluid (TF) and lacrimal fluid (LF) (dry eye disease [DED] vs. control) were isolated individually and tryptic peptides were separated into 24 fractions and concatenated into 6 fractions by high-pH reversed-phase liquid chromatography fractionation. The resulting peptides were desalted and analysed by liquid chromatography mass spectrometry (LC-MS/MS) followed by MaxLFQ quantification at the MS-1 level. **(b)** Diagram describing our approach for identifying LF proteins and comparison to TF proteins. In total, 1764 proteins were identified, and 849 proteins (48%) were present in both fluids. **(c)** Cellular components of identified proteins in TF and LF. Y-axis shows gene count. Red stars mark the extracellular region, and blue stars mark the cytosol.

The proteins in pooled TF and LF samples from patients with DED (n = 5) and controls (n = 5) were analysed by liquid chromatography MS (LC-MS/MS) following trypsin digestion and high-pH reverse-phase liquid chromatography fractionation (Fig. 1a, Table 1). In total, 1165 proteins and 1448 proteins were identified in the TF and LF with a false discovery rate of less than 1% at the protein and peptide spectrum match levels, respectively. In total, 1764 proteins mapped to 1671 genes were identified in TF and LF samples (Supplementary Table S1).

**Table 1:** Clinical parameters for the classification of dry eye disease and control patients at each proteomic analysis.

Specific step of proteomics analysis	Group	No. of Patients (female, n)	Age (years)	Schirmer test I (mm)	Tear breakup time (s)	Protein concentration (μg/μl)
Global profiling	DED	5 (4)	62.20 ± 8.67	5.80 ± 1.30	3.40 ± 1.14	TF: 11.86 ± 2.10
						LF: 11.02 ± 1.04
	Control	5 (4)	61.80 ± 7.79	13.80 ± 1.64	8.20 ± 0.84	TF: 9.79 ± 0.84
						LF: 10.59 ± 1.90
MRM assay	DED	17 (13)	60.92 ± 9.01	5.76 ± 1.52	3.58 ± 0.87	TF: 13.06 ± 1.81
						LF: 11.44 ± 1.19
	Control	17 (13)	61.06 ± 7.91	11.47 ± 2.00	7.89 ± 1.26	TF: 11.78 ± 4.90
						LF: 11.69 ± 3.69

Data are means ± standard deviation; MRM, Multiple reaction monitoring; DED, dry eye disease; TF, tear fluid; LF, lacrimal fluid.

Among the identified proteins in TF and LF, only 48% (489) were common between TF and LF, indicating that 599 proteins in LG were not detected in TF. In addition, 316 proteins not found in LF were identified in TF. Therefore, these proteins originated from the ocular surface (Fig. 1b).

To determine the cellular components of TF and LF proteins, we conducted Gene Ontology (GO) analysis using Database for Annotation, Visualization and Integrated Discovery (version 6.7). Figure 1c demonstrates that the cellular pool of TF comprised mostly extracellular region proteins such as defensin β1 (DEFB1), tenascin XB (TNXB), N-acetylgalactosaminyltransferases 1 (GALNT1), and etc., whereas that of LF consisted largely of cytosol proteins such as ribosomal proteins (RPL18, RPLP1, RPS16), histidyl-TRNA synthetase (HARS), adenylosuccinate Lyase (ADSL) and so on. Notably, proteins specifically identified in each fluid showed distinct biological characteristics. TF-specific proteins (In total, 316) belonged to the oligosaccharide metabolic process and glycosylation-related processes groups, whereas LF-specific proteins (In total, 599) belonged to the translation and RNA processing groups (Supplementary Fig. S1).

### Differentially expressed proteins in the TF and LF of patients with DED

We found 138 differentially expressed proteins (DEPs) in TF and 161 DEPs in LF (fold-change > 2) (Supplementary Table S2). Among the proteins, PLA2G2A, PCBP1, and YWHAB were simultaneously upregulated and UBA52, CALML5, CEACAM1, and WFDC were downregulated in both the TF and LF of DED patients. Furthermore, the expression levels of FGB, IGHM, and GANAB were increased in LF but reduced in TF in DED patients compared to in controls. In contrast, expression levels of LAP3, SERPINB2, CTSL, and ZG16B were increased in TF but reduced in LF in DED. When all quantifiable proteins were considered, approximately 50% of the DEPs in TF and LF between DED and control samples showed equivalent increasing or decreasing patterns. Supplementary Fig. S2 shows the alteration patterns of proteins with protein-protein interaction information from the Search Tool for the Retrieval of Interacting Genes (STRING) database and were enclosed by GO-BP (Biological Process) terms.

Next, we compared our data for proteins types with those identified in four previous studies of DED biomarkers from TF based on an fold-change > 1.5^16–19^. Of the 77 TF proteins whose expressions were altered by DED, as reported in previous studies, 37 showed a consistent alteration pattern in this study. The expression pattern of five downregulated proteins in TF, including defensin α family (DEFA1) and lactoperoxidase (LPO), corresponded to previously reported results. Furthermore, 11 upregulated proteins in this study were previously reported to be upregulated in TF as the result of DED. Interestingly, LF DEPs showed alteration patterns consistent with the reported expression patterns of DEPs in previous TF proteomics studies. Among them, 20 upregulated [e.g., a-2-HS-glycoprotein (AHSG), valosin-containing protein (VCP), and orosomucoid 1 (ORM1)] and 7 downregulated proteins [lipocalin-1 (LCN1), alpha-2-glycoprotein 1, zinc-binding (AZGP1), and lysozyme C (LYZ)] showed the same quantitative patterns as those reported in previous TF proteomics studies^23^ (Supplementary Tables S3a,b).

### Gene ontology analysis revealed a key regulator of DED function

**Figure 2:**
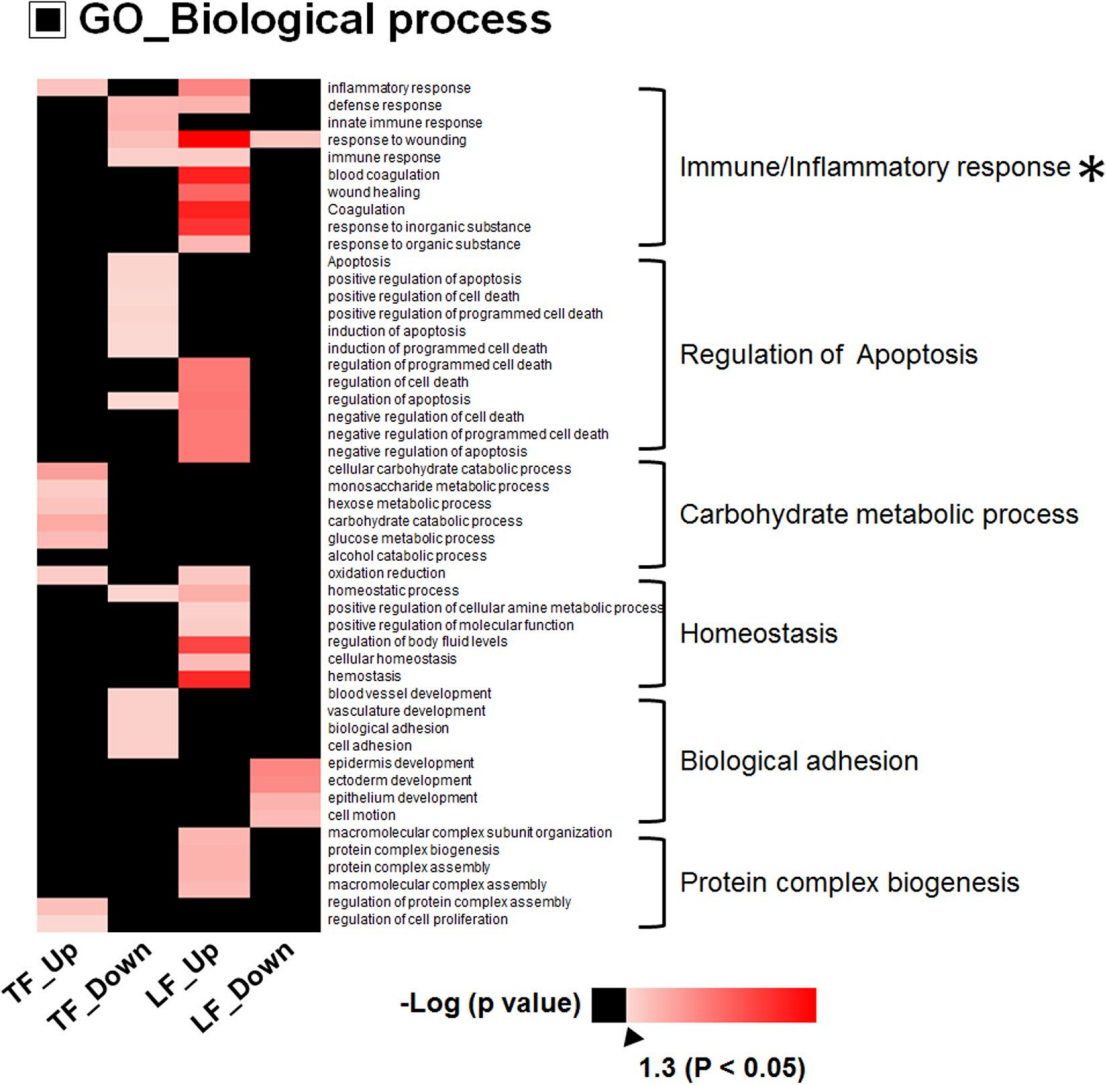
Gene Ontology analysis of differentially expressed proteins in tear fluid and lacrimal fluid. Heatmap showing significantly represented Gene Ontology (GO) biological process terms (p < 0.05) for differentially expressed proteins between dry eye disease and control samples. The red colour in the heatmap indicates a significant alteration of the biological process. The asterisk marks the most enriched GO category in the heatmap.

To gain insight into the functional roles of DEPs associated with DED in TF and LF, we first compared the GO-BP and Kyoto Encyclopedia of Genes and Genomes (KEGG) pathway analysis using up- and downregulated DEPs (Supplementary Tables S4a–d, S5a–c). Next, we displayed the GO-BP heatmap to detect alterations at the molecular systems level of the proteome associated with DED. Forty-nine categories were enriched and are presented in the heatmap (Fig. 2).

Notably, the GO-BP category of ‘immune/inflammatory response process’ was actively changed in LF, but not in TF. Most upregulated LF proteins, including complement C4b (C4B), haptoglobin (HP), Alpha-1-acid glycoprotein 1 (ORM1), and clusterin (CLU), belonged to the GO-BP category of ‘immune response’ from only LF. Moreover, the category of ‘apoptosis regulation’ was down-regulated in TF and up-regulated in LF, indicating that the cell death and protective mechanism from immune-inflammatory cytokines is more active in LG than on the ocular surface. Additionally, carbohydrate mechanism-related genes were up-regulated in TF, but not in LF.

### Proteome interaction network model describing DED

**Figure 3:**
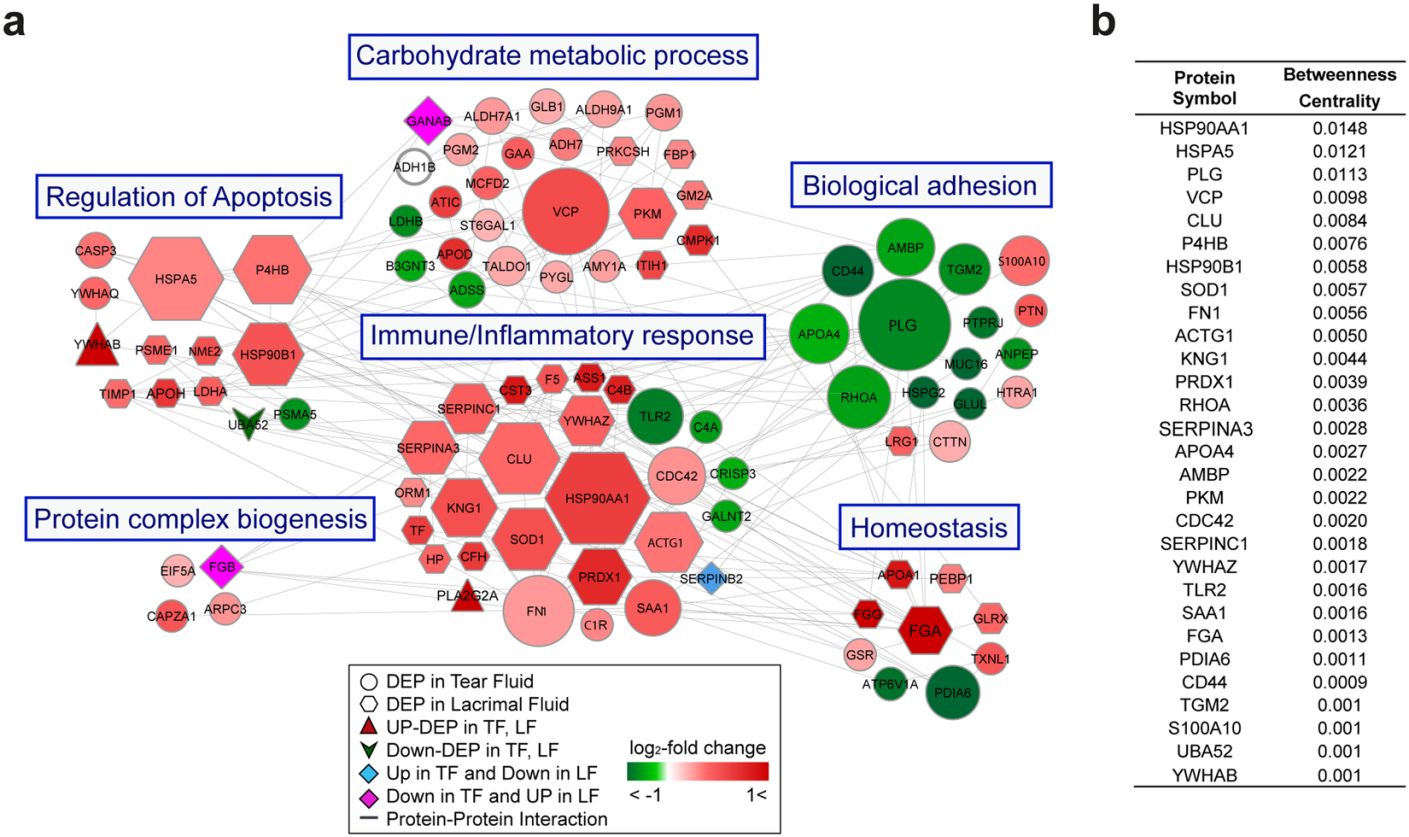
Network modelling of regulated fluid proteins in dry eye disease versus control samples. (**a**) Network model showing the biological processes affected, including the immune/inflammatory response, carbohydrate metabolic process, biological adhesion, regulation of apoptosis, homeostasis, and protein complex biogenesis. The colours of the nodes represent proteins whose levels were greatly increased (red) or decreased (green) in dry eye disease. Each shape represents a distinguishable alteration condition (outlined in the box). The connection between nodes (grey lines) shows either a regulatory role or physical interaction between proteins. Large nodes represent a high degree of connectivity with other proteins in the network. **(b)** List of the top 29 proteins based on betweenness centrality (>0.001).

To prospectively understand the map of cellular networks altered in the TF and LF of DED patients, we constructed network models using DEPs from TF and LF by using STRING database information (Fig. 3a). From this map, we explored the key proteins involved in crucial biological processes that may influence the pathogenesis of DED by carrying out a calculation of centrality (weighted closeness) based on the degree of betweenness (Fig. 3b). The results included the top 29 proteins based on degree and betweenness centrality which reflect the amount of control that this node exerts over the interactions of other nodes in the network^24^. Heat shock protein HSP 90-alpha (HSP90AA1) showed the highest betweenness centrality in this network model. Additionally, these proteins formed a close network with the category of ‘apoptosis’, or ‘homeostasis’ and showed strong centrality within the protein-protein network model. The proteins were related to the innate immune response, specifically the complement pathway, and were activated systematically in our DED network model.

### Verification of candidate biomarkers by multiple reaction monitoring

**Figure 4:**
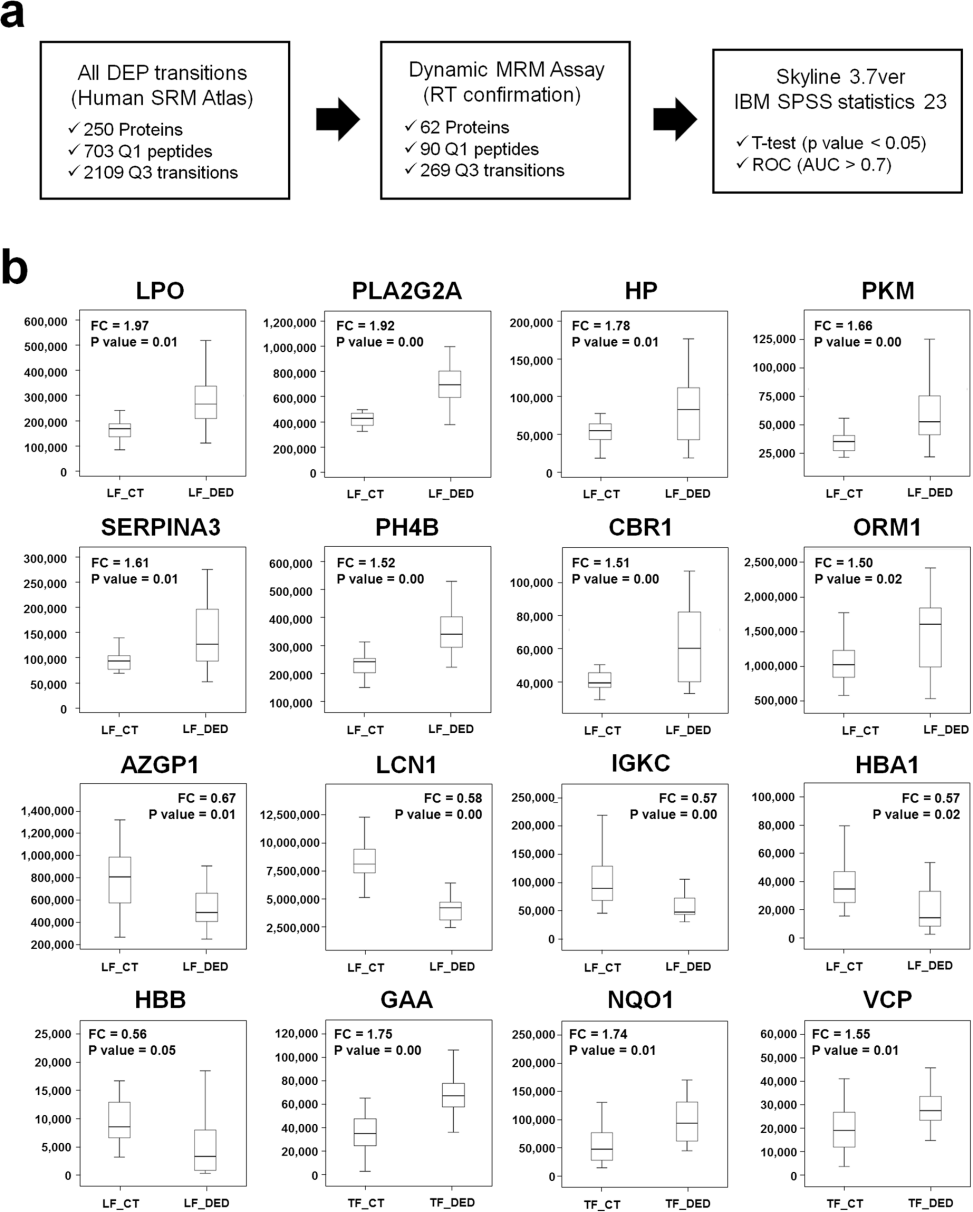
Investigation of candidate biomarkers using multiple reaction monitoring. (**a**) Overall work flow of the multiple reaction monitoring assay (MRM). MRM target transitions (703 (Q1), 2109 (Q3)) were selected from Human SRMAtlas resource followed by verifying the detectability and retention time information. Final candidate markers (90 (Q1), 269 (Q3)) were analysed by dynamic MRM assay in triplicate. *t*-Test and receiver operating characteristic curve between dry eye disease (DED) and control (CT) samples conducted using SPSS version 21.0 (SPSS, Inc., Chicago, IL, USA). **(b)** Interactive box plots of 16 DED candidate markers (3 in TF and 13 in LF). These proteins showed alteration patterns that were consistent with LFQ data. The fold-change and *p*-value for each marker are indicated on the interactive box plots.

We performed MRM analysis using 100 μg proteins from DED patients [TF, n = 17; LF, n = 17] and controls [TF, n = 17; LF, n = 17] to verify the DEPs in the LFQ data (Table 1). As a result, 62 proteins were confirmed as candidates and 269 Q3 transitions were generated (Fig. 4a, Supplementary Table S6). To verify the reproducibility of the LC-MRM-MS runs, we evaluated coefficients of variation between triplicate results based on the best transitions of each peptide. The overall median and average coefficients of variation of the target peptides were 6.7% and 11.7%, respectively. Based on MRM quantification, 11 and 24 proteins were significantly changed in the TF and LF of DED compared to controls (p value < 0.05, FC > 1.5), respectively (Supplementary Table S7). Among these proteins, 3 up-regulated proteins in TF (GAA, NQO1, and VCP) showed consistent alteration patterns as in the LFQ quantification set. Furthermore, 8 up-regulated proteins (LPO, PLA2G2A, HP, PKM, SERPINA3, P4HB, CBR1 and ORM1) and 5 downregulated proteins (IGKC, LCN1, HBA1, HBB, and AZGP1) in LF were consistent with the LFQ DEPs. Their alteration patterns are represented as interactive box plots which show that the marker candidates could clearly discriminate between DED patients and controls (Table 2 and Fig. 4b).

**Table 2:** Differentially expressed marker proteins obtained from MRM assay to be consistent with DEPs in LFQ data. (* Fold change > *1.5, p value* < *0.05, AUC* > *0.7).*

Protein name	Gene Name	Peptide sequence	fold change (DED/CT)	p value (T-test)	AUC	Consistent alteration
Lysosomal alpha-glucosidase	GAA	GVFITNETGQPLIGK	1.75	0.00	0.88	*Up in TF*
NAD(P)H dehydrogenase [quinone] 1	NQO1	ALIVLAHSER	1.74	0.01	0.79	*Up in TF*
Transitional endoplasmic reticulum ATPase	VCP	VINQILTEMDGMSTK	1.55	0.01	0.78	*Up in TF*
Lactoperoxidase	LPO	IHGFDLAAINTQR	1.97	0.01	0.82	*Up in LF*
Phospholipase A2, membrane associated	PLA2G2A	FSNSGSR	1.92	0.00	0.88	*Up in LF*
Haptoglobin	HP	VTSIQDWVQK	1.78	0.01	0.71	*Up in LF*
Pyruvate kinase PKM	PKM	IYVDDGLISLQVK	1.66	0.00	0.80	*Up in LF*
Alpha-1-antitrypsin	SERPINA3	ADLSGITGAR	1.61	0.01	0.74	*Up in LF*
Protein disulfide-isomerase	P4HB	VDATEESDLAQQYGVR	1.52	0.00	0.88	*Up in LF*
Carbonyl reductase [NADPH] 1	CBR1	IGVTVLSR	1.51	0.00	0.71	*Up in LF*
Alpha-1-acid glycoprotein 1	ORM1	YVGGQEHFAHLLILR	1.50	0.02	0.70	*Up in LF*
Zinc-alpha-2-glycoprotein	AZGP1	QDPPSVVVTSHQAPGEK	0.67	0.01	0.75	*Down in LF*
Lipocalin-1	LCN1	VTMLISGR	0.58	0.00	0.83	*Down in LF*
Ig kappa chain C region	IGKC	VDNALQSGNSQESVTEQDSK	0.57	0.00	0.79	*Down in LF*
Hemoglobin subunit alpha	HBA1	MFLSFPTTK	0.57	0.02	0.78	*Down in LF*
Hemoglobin subunit beta	HBB	EFTPPVQAAYQK	0.56	0.05	0.76	*Down in LF*

**Figure 5:**
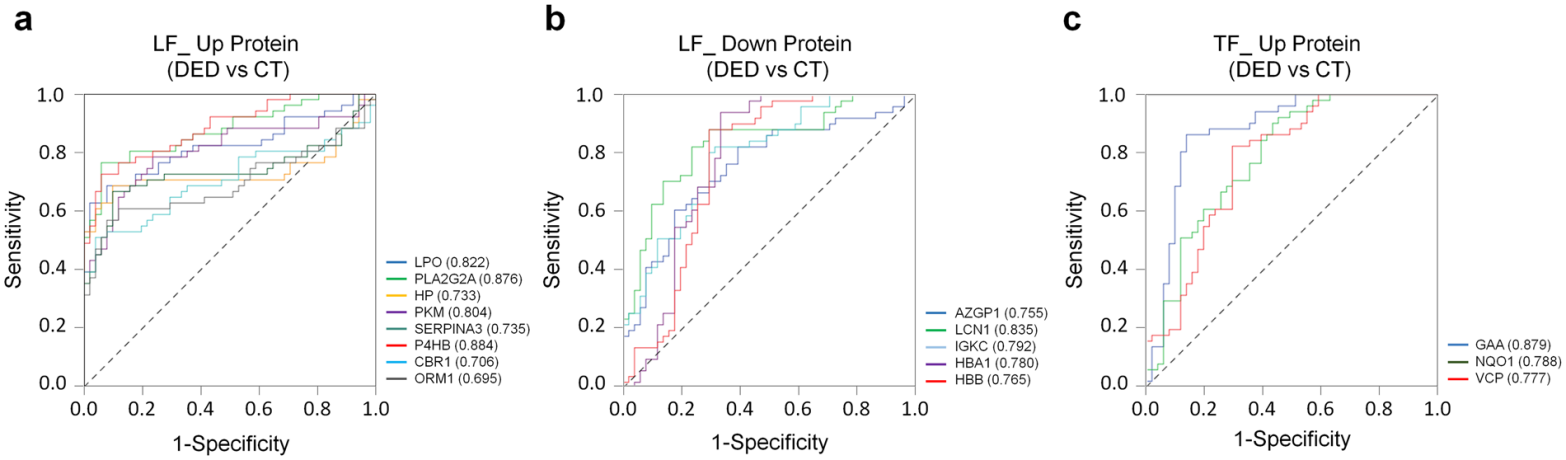
Verification of novel biomarkers for dry eye disease by comparison of receiver operating characteristics curves in tear fluid and lacrimal fluid with dry eye disease. (**a**) Eight marker proteins were up-regulated and (**b**) 5 were down-regulated in lacrimal fluid. In addition, (**c**) 3 proteins in tear fluid were upregulated. All novel markers showed high sensitivity and selectivity with an area under the curve of greater than 0.7.

To confirm the sensitivity and selectivity of the 16 candidate markers for DED, we calculated the area under the receiver operating characteristic curves (AUC). All marker proteins showed high AUC values exceeding 0.7, as shown in Fig. 5. Particularly, the LG-secreted proteins LPO and PLA2G2A which are related to the immune/inflammatory response showed very high sensitivity and specificity with AUC values of 0.822 and 0.876, respectively, in DED patients.

## Discussion

There were three important findings in this study. First, we isolated and identified nearly 1700 proteins from ocular surface fluid, while only several hundred proteins have been previously documented in proteomics analyses of human TF^16–19^. Therefore, this study represents the largest number of ocular surface fluid proteins of DED patients identified to date. Second, this is the first study reporting the proteomics of human LF compared to those of TF. With the development of LF collection methods, we directly collected fluid originating from the LG, identified and measured protein and expression levels, and compared them with TF proteomics data. Accordingly, the most important pathway and proteins among the DEPs were in the categories of ‘immune/inflammatory response from LF, not from TF. Finally, after the DEPs of TF and LF were discriminated between DED patients and controls, they were validated by an MRM assay in larger individual samples. Thus, we identified novel candidate markers with high sensitivity and specificity for DED patients.

In this study, we identified a larger number of proteins in LF than in TF (1448 vs. 1165), although TF contains a greater number of different cells such as the corneal epithelium, immune cells, nerves, vascular cells, Goblet cells, and meibomian gland cells. Moreover, approximately half of the identified proteins (849) were common between TF and LF. These results indicate that 316 TF proteins were not secreted from the LG and that the LG contributes approximately three-quarters of the TF protein composition. Interestingly, 599 proteins secreted from the LG were not found in the TF. Although the fate of these proteins was not determined in the present study, there may be two possibilities explaining their loss. These proteins may be used on the ocular surface soon after secretion from the LG or they are easily degraded by proteases expressed on the ocular surface and/or TF. In addition, a relatively large number of proteins from the corneal/conjunctival epithelium is thought to be secreted under DED conditions.

Our GO-BP and KEGG analyses revealed that the most abundant TF-proteins belonged to the extracellular region. Specifically, DEFB1 is well known as antimicrobial peptides produced by corneal and conjunctiva as well as lacrimal gland^25^. TNXB is thought to function in wound healing process during matrix insult like desiccating stress on the ocular surface. Also, GALNT1 is one of the important enzymes involved in the initiation of mucin-type O-glycosylation in human conjunctiva^26^. On the other hand, LF-proteins predominantly belonged to the cytosol area. Since LGs are exocrine glands that actively synthase and secrete proteins with aqueous layer of tears, LF contains ribosomal proteins (e.g., RPL18, RPLP1, RPS16) and HARS which play an essential role in catalysing protein biosynthesis. ADSL is also important in sustaining metabolic and energetic nucleotide cycles for active exocytosis^27^. Collectively, LF and TF proteins originate from different pools and other types of cells, therefore, have some different physiological functions, although >800 proteins overlapped between the two fluids in the present study.

We found that several known biomarker proteins in the TF showed similar expression patterns in the LF of DED patients. In particular, reductions of LCN1, LTF, and LYZ, which are related to the immune response in TF, have been well-documented in previous studies^18,28^. The authors suggested that their decrease in tears is a relevant indicator of LG dysfunction^18,28,29^. According to this study, down-regulation of these marker proteins is caused by proteomic changes in the LF in DED, resulting in impairment of the bacterial defence system in the LF environment. This may render patients more susceptible to microorganism growth on the ocular surface. The expression levels of these proteins were previously shown to decrease only in TF, but our results demonstrated that they are also decreased at the LF level.

In this study, we verified the DEPs in an MRM assay, which is a highly sensitive and selective method for targeted quantification of protein abundance^30^. To select proper MRM transitions, we utilized Human SRMAtlas^31^, which provides definitive and verified peptide transitions and collision energy information optimized by quadrupole-based mass spectrometry. Furthermore, the Agilent Bravo Platform, which uses an accurate liquid handling system, provided highly reproducible preparation for 68 individual samples. The MRM assay revealed, in total, 16 marker proteins (3 in TF and 13 in LF) that were consistent with the LFQ quantification results. In addition, these proteins showed very high AUC values of greater than 0.7 (Fig. 5) and may be novel biomarkers for discriminating between DED patients and controls. Of these proteins, immune response-related proteins in the LF including PLA2G2A, LPO^32^, HP^33^, SERPINA3 and ORM1 were significantly increased in both the LFQ and MRM data. Especially, in the MRM verification set, PLA2G2A was significantly up-regulated in LF (fold-change = 1.92, *p*-value = 0.0015, AUC = 0.88), but not in TF. PLA2G2A is a pro-inflammatory enzyme that catalyses the initial step of the arachidonic acid pathway^34^. Moreover, PLA2G2A in TF plays a major role in killing a broad spectrum of gram-positive and gram-negative bacteria at the ocular surface under physiological conditions^35,36^. In previous studies, the ocular surface was found to show increased levels of PLA2GA in the TF of DED patients and DED mouse conjunctival tissue^37,38^. Although this biomarker candidate was previously found to be important in preventing microbial infections at the ocular surface under DED conditions, our results revealed that it is highly activated in the LF as well. Additionally, LCN1 was significantly down-regulated in the LF of DED patients (AUC = 0.83). This indicates that immune response-related proteins known to be biomarkers in TF are more meaningful marker proteins in LF.

Notably, innate immune defence-related proteins, specifically those related to the complement pathway, were up-regulated at the protein level in LF. Especially, LPO, C4B, F5, FGG, FGA, FGB, KNG1, MIF, SERPINC1, SERPING1, SERPINA1, SERPINA3 and PRDX1 were increased in the LF of DED patients in LFQ data. The complement pathway has not been investigated previously in either non-Sjögren syndrome or Sjögren syndrome DED. Complement pathway proteins are now thought to be important markers of age-related macular degeneration^39,40^, bacterial keratitis^41^, and ocular allergy^42^.

In this study, we could not compare the expression patterns at the gene level in certain related human tissues including the LG and cornea. It is difficult to carry out human LG biopsies because of the LG’s highly vascularized structures and ethics limitations. However, valuable information may be obtained when transcriptomic and proteomic analyses are performed with human ocular tissues in future studies. In addition, another limitation of this study was the small number of cohort samples. Although we identified 16 meaningful candidate marker proteins for DEP through both LFQ and MRM analysis, further studies including more TF and LF samples from DED patients and control groups are needed to apply these markers in diagnosis.

In conclusion, we provide fundamental information regarding biomarker candidates for DED using proteomics of fluids from the human ocular surface and subsequent MRM verification in individual samples. Because there are no definitive diagnostic criteria or biomarkers for DED to date, the identification of biomarkers in DED may lead to more accurate diagnosis and grading of DED and ultimately to the development of targeted drug therapies. Further studies of larger patient cohorts are needed to determine and select more accurate markers representing the DED pathology, determine drug selection, and evaluate disease prognosis.

## Materials and Methods

### Patient enrollment and determination of ocular surface dryness

This cross-sectional, case-control clinical trial was conducted at two sites: Gangnam Severance Hospital (Department of Ophthalmology, Yonsei University College of Medicine, Seoul, Korea) and Sacred Heart Hospital (College of Medicine, Hanllym University, Chuncheon, Gangwon-Do, Korea). All procedures conformed to the tenets of the Declaration of Helsinki. The study was approved by the Institutional Review Board of each hospital, and informed consent was obtained from all patients. DED was diagnosed according to the diagnostic criteria of Asia Dry Eye Society^43^. The inclusion criteria were as follows: one or more DED-related symptoms, including tightness, foreign body sensation, irritation, red-eye, itching sensation, blurring, or pain; a Schirmer’s test I result (without anesthesia) of <5 mm in 5 minutes, a tear break-up time of <5 seconds or a typical DED pattern of superficial punctuate erosion of the conjunctiva or cornea. Patients were excluded if they had (1) a history of using eye drops within the current month, (2) infection, trauma, an ocular procedure, or other surgery within the previous 6 months, (3) severe blepharitis with meibomian gland dysfunction, (4) a blinking abnormality (e.g., Parkinson’s disease or facial nerve palsy), or (5) severe pterygium or an uncontrolled systemic disease. Pregnant or lactating patients were also excluded. Clinical tests for DED and human fluid-sampling of TF and LF were performed by an ophthalmologist (H.S.H). The other author (Y.W.J) collected the fluids and prepared them for analysis. The data were then analyzed by another author (E.J.C). All evaluations were performed in a blinded fashion on the disease status of all subjects. Table 1 summarizes the clinical parameters for the classification of DED and control samples at each step of proteomic analysis including global profiling and MRM assay.

### Sampling of tear fluid and lacrimal fluid for proteomics

To measure and collect the fluids from patients’ tears, a bonded 2.0 × 10-mm polyester fiber rod (TRANSORB® WICKS, FILTRONA, Richmond, VA, USA) was used as previously reported. Briefly, to collect the TF which is a mixed fluid of the secretion from the LGs, meibomian glands, and corneoconjunctival cells, a polyester wick was applied to the tear meniscus of the lower lid margin. After then, it was removed and placed into a 1.5-ml Eppendorf tube, which was stored at −70 °C until the mass spectrophotometric assay was performed. In addition to the TF, pure LF was collected. The detailed methods for collecting TF and LF were published with video file^44^.

### In-solution digestion

100 μg Proteins from pooled TFs and LFs (n = 5 for each fluid and experimental group) were digested into peptides by in-solution digestion. 8 M urea in 100 mM ammonium bicarbonate (Sigma, St. Louis, MO, USA) was mixed with each fluid sample at a 1:1 ratio, and the mixture was incubated for 20 min at room temperature (RT). Then, 10 mM DTT (Dithiothreitol, Sigma) for reduction and 30 mM IAA (Iodoacetamide, Sigma) for alkylation were used to denature the proteins. Trypsin was added to the samples (1:50 = trypsin:sample) and incubated at 37 °C overnight. The activated trypsin reaction was quenched with 0.4% TFA, and peptides were desalted with a C18 Harvard macro spin column. The resultant peptides were dried and stored at −80 °C.

### High-pH reverse-phase liquid chromatography (RPLC) fractionation

Peptide separation was carried out using high-pH RPLC fractionation based on peptide hydrophobicity. Samples were divided into six fractions using the Agilent 1260 series HPLC system (Agilent Technologies, Santa Clara, CA, USA). Briefly, an Accucore™ 150 C18 LC column (150 mm × 2.1 mm, 4 μm) was used for fractionation with the high-pH buffer A, B; 10 mM ammonium formate (pH = 10) as mobile phase A and 10 mM ammonium formate in 90% ACN (pH = 10) as mobile phase B. The gradient was as follows: 0–10 min, 5% B; 10–70 min, 35% B; 70–80 min, 70% B; 80–85 min, 70% B; 85–90 min, 5% B; 90–105 min, 5% B. The separated peptides were collected and dried in a speed-vac. Each fraction was desalted with a C18 spin column.

### Protein identification using LC-MS/MS

Peptides fractionated into six fractions were re-suspended in 0.1% Formic Acid (FA) in water and analyzed using the Q ExactiveTM Orbitrap Hybrid Mass Spectrometer coupled with the EASY-nLC 1000 system (ThermoScientific, Bremen, Germany). For the proteome profiling analysis, the gradient was as follows: from 5% to 40% of solvent B for 130 min, from 40% to 80% of solvent B for 5 min, holding at 80% of solvent B for 10 min, and equilibrating the column at 1% of B for 30 min (Sol A: 0.1% FA in water, Sol B: 0.1% FA in Acetonitrile). The peptides were eluted through a trap column, ionized through an EASY-spray column (50 cm × 75 μm ID) packed with 2 μm C18 particles at an electric potential of 1.8 kV. Full MS data were acquired in a scan range of 400–2,000 Th at a resolution of 70,000 at m/z 200, with an automated gain control (AGC) target value of 1.0 × 10^6^ and a maximum ion injection of 120 ms. The maximal ion injection time for MS/MS was set to 60 ms at a resolution of 17,500. Dynamic exclusion time was set to 30 s.

### Raw data processing

The MS2 spectra were searched with the MaxQuant (v. 1.5.1.2)^45^ against the Uniprot human database (released in June, 2014). Carbamidomethylation of cysteine as a fixed modification and N-acetylation and oxidation of methionine as variable modifications were used for each search. A false discovery rate (FDR) cutoff of 1% was applied at the peptide spectrum match (PSM) and protein levels. An initial precursor mass deviation up to 4.5 ppm and a fragment mass deviation up to 20 ppm were allowed. Protein identification required at least one peptide using the ‘razor plus unique peptides’ setting in MaxQuant^46^. Proteins were quantified using the XIC-based label-free quantification (LFQ) algorithm in MaxQuant^47^. The ‘match between runs’ option was used for nonlinear retention time alignment. The match time window was 0.7 min, and the alignment time window was 20 min.

### Analysis for proteomics data

Further statistical and bioinformatics analyses were performed using Perseus software (v. 1.5.0.31). Proteins that were quantified by at least two peptides using the ‘unique plus razor peptides’ setting in MaxQuant were used for LFQ to prevent ambiguous abundance comparison. Before loading the LFQ intensity data, hits to the reverse database, contaminants, and proteins only identified by site were eliminated. After loading the data, all duplicate data were grouped separately. All LFQ intensities were transformed to log2 values. Proteins that did not display all values in at least one group were filtered out. Additionally, in cases with a missing value, missing values were replaced by imputation based on the normal distribution (using a width of 0.3 and a downshift of 1.8)^48^. Proteins with expression greater than ±2-fold change from Student’s t-test in LFQ intensity were classified to differentially expressed proteins (DEPs). Further annotation enrichment analyses were performed on the resulting significantly DEPs.

### Enrichment analysis using gene ontology and network analysis

A gene ontology (GO) search was performed to explore the biological processes and cellular components in TF and LF associated with DED. KEGG pathway mapping was also performed using DAVID freeware^49^. GO biological processes enriched by the DEPs were identified as those with a p-value < 0.05. To construct a network depicting the enriched processes, we selected DEPs involved in enriched biological processes. To reconstruct the network model for DEPs, we collected protein-protein interactome information from the STRING 9.1 public database^50^. The network model was built with sorted DEPs and interactome data using Cytoscape.

### Automated Peptide Sample Preparation for LC-MRM analysis

Sample preparation for LC-MRM including tryptic digestion and peptide cleanup were automated using Assay Map bravo platform from Agilent technologies with VWorks Automation Control 11.1 software^51^. A total of 68 samples (Control_TF (17), DED_TF (17), Control_LF (17), DED_LF (17)) were digested reconstituted with a 8 M urea in 50 mM ABC buffer (pH 8.0). Protein was reduced with 10 mM DTT for 30 min at 37 °C and was alkylated by blocking the cysteine residues using 40 mM IAA at RT for 1 h in dark. Urea concentration was reduced to below 1 M using 50 mM ABC buffer. The proteins were digested using sequence graded trypsin at 50:1 ratio for protein: trypsin at 37 °C overnight. Each sample in 96 well plate was acidified by the addition of 10% TFA and the pH was reduced to 2–3 before desalting. The acidified digests was immediately processed through the Peptide Cleanup Protocol using Sep-Pak C18 96-well plate (100 mg Sorbent per well, Waters). The eluted peptides with 80% acetonitrile (ACN) were dried by speed vaccum.

### Transition selection and LC-MRM analysis

(1). Unique peptides in human database were selected for representing target proteins with following criteria: fully tryptic peptides with no missed cleavages, unique to a particular protein, with a length between 6 and 30 amino acids. To quantify the proteins, at least two peptides are selected and three product ions were selected. Optimized collisional energy from SRMAtlas database^31^ was applied in our dynamic MRM (dMRM) analysis. To validate the existence of target transitions, selected transitions of target proteins were tested with several MRM scans and transitions which have peaks higher than 1000 area were enriched as final transitions. Final transitions were analyzed using digested peptides in dMRM mode. RP-HPLC column (150 × 2.1 mm ID, Agilent Zorbax Eclipse Plus C18 Rapid Resolution HD, 1.8 um particles) equipped with Agilent 1290 LC separated digested peptides and Agilent 6490 triple-quadrupole mass spectrometer, controlled by Agilent’s MassHunter Workstation software (v.B.06.00), generated dMRM result data. Gradient was set up as below: HPLC gradient started at 5% solvent B (90% ACN, 0.1% formic acid) for 2 min and went up to 30% solvent B during a 36 min time period and raised up to 40% solvent B in 4 min, followed by a steep increase to 80% B within 2 min. After retaining 80% solvent B at 4 min, equilibrium was held with 5% sol B 12 min A post column equilibration time of 4 min was used after every sample analysis. Following parameters were used for MRM acquisition: 3500 V capillary voltage, 300 V nozzle voltage, 11 L/min sheath gas flow at a temperature of 250 °C (ultra-high-purity nitrogen),15 L/min drying gas flow at a temperature of 150 °C (ultra-high-purity nitrogen), 30 psi nebulizer gas flow (ultra-high-purity nitrogen), 380 V default fragmentor voltage, 5 V cell accelerator potential, and wide (1.2 da full-width-at-half-maximum) and unit resolution (0.7 Da full-width-at-half-maximum) in the first and third quadrupoles, respectively. To gain enough number of dot points, dwell times for each transitions were determined between 6.55 and 248.88 ms, with transitions being the maximum number that could be monitored in a given 1000-ms cycle.

### Data Analysis and Statistics

The areas were extracted using Skyline 3.7ver^52^. Savitzky−Golay smoothing was applied to increase the quality of the chromatograms. Peptides’ areas between 204 runs were normalized to ß-galactose peptide (APLDNDIGVSEATR, 729.36 m/z (Q1) → 563.28 m/z (Q3), CV = 16.58%) to correct experimental variation. The best transition was selected on the basis of intensity and consistency for the quantification. Independent t-test, ROC (receiver operating characteristic) analysis was conducted using SPSS version 21.0 (IBM Corp., Armonk, NY, USA) to determine the significance of target proteins.

## Electronic supplementary material

Supplementary Information

Supplementary Table S1.

Supplementary Table S2.

Supplementary Table S3.

Supplementary Table S4.

Supplementary Table S5.

Supplementary Table S6.

Supplementary Table S7.

## References

[1] Grus FH, Joachim SC, Pfeiffer N (2007). Proteomics in ocular fluids. Proteomics Clin Appl.

[2] Hu S, Loo JA, Wong DT (2006). Human body fluid proteome analysis. Proteomics.

[3] Agnifili L (2015). Molecular biomarkers in primary open-angle glaucoma: from noninvasive to invasive. Prog Brain Res.

[4] von Thun Und Hohenstein-Blaul N, Funke S, Grus FH (2013). Tears as a source of biomarkers for ocular and systemic diseases. Exp Eye Res.

[5] Boehm N (2013). Alterations in the tear proteome of dry eye patients–a matter of the clinical phenotype. Invest Ophthalmol Vis Sci.

[6] Boehm N, Riechardt AI, Wiegand M, Pfeiffer N, Grus FH (2011). Proinflammatory cytokine profiling of tears from dry eye patients by means of antibody microarrays. Invest Ophthalmol Vis Sci.

[7] Matheis N, Okrojek R, Grus FH, Kahaly GJ (2012). Proteomics of tear fluid in thyroid-associated orbitopathy. Thyroid.

[8] Herber S, Grus FH, Sabuncuo P, Augustin AJ (2002). Changes in the tear protein patterns of diabetic patients using two-dimensional electrophoresis. Adv Exp Med Biol.

[9] Bohm D (2012). Comparison of tear protein levels in breast cancer patients and healthy controls using a de novo proteomic approach. Oncol Rep.

[10] Lebrecht A (2009). Diagnosis of breast cancer by tear proteomic pattern. Cancer Genomics Proteomics.

[11] Lebrecht A, Boehm D, Schmidt M, Koelbl H, Grus FH (2009). Surface-enhanced Laser Desorption/Ionisation Time-of-flight Mass Spectrometry to Detect Breast Cancer Markers in Tears and Serum. Cancer Genomics Proteomics.

[12] Schein OD, Munoz B, Tielsch JM, Bandeen-Roche K, West S (1997). Prevalence of dry eye among the elderly. Am J Ophthalmol.

[13] Schein OD, Tielsch JM, Munoz B, Bandeen-Roche K, West S (1997). Relation between signs and symptoms of dry eye in the elderly. A population-based perspective. Ophthalmology.

[14] Smith RE (2005). The tear film complex: pathogenesis and emerging therapies for dry eyes. Cornea.

[15] Uchino M (2011). Prevalence and risk factors of dry eye disease in Japan: Koumi study. Ophthalmology.

[16] Li B (2014). Tear proteomic analysis of patients with type 2 diabetes and dry eye syndrome by two-dimensional nano-liquid chromatography coupled with tandem mass spectrometry. Invest Ophthalmol Vis Sci.

[17] Li B (2014). Tear proteomic analysis of Sjogren syndrome patients with dry eye syndrome by two-dimensional-nano-liquid chromatography coupled with tandem mass spectrometry. Sci Rep.

[18] Zhou L (2009). Identification of tear fluid biomarkers in dry eye syndrome using iTRAQ quantitative proteomics. J Proteome Res.

[19] Soria J (2013). Tear proteome and protein network analyses reveal a novel pentamarker panel for tear film characterization in dry eye and meibomian gland dysfunction. J Proteomics.

[20] Kim, E. C. *et al*. Direct visualization of aqueous tear secretion from lacrimal gland. *Acta Ophthalmol*; 10.1111/aos.13335. (2016). 10.1111/aos.13335 27879057

[21] Blalock TD, Spurr-Michaud SJ, Tisdale AS, Gipson IK (2008). Release of membrane-associated mucins from ocular surface epithelia. Invest Ophthalmol Vis Sci.

[22] Santacruz C (2015). Expression of IL-8, IL-6 and IL-1beta in tears as a main characteristic of the immune response in human microbial keratitis. Int J Mol Sci.

[23] Versura P (2010). Tear proteomics in evaporative dry eye disease. Eye (Lond).

[24] Yoon J, Blumer A, Lee K (2006). An algorithm for modularity analysis of directed and weighted biological networks based on edge-betweenness centrality. Bioinformatics.

[25] Haynes RJ, Tighe PJ, Dua HS (1999). Antimicrobial defensin peptides of the human ocular surface. Br J Ophthalmol.

[26] Guzman-Aranguez A, Mantelli F, Argueso P (2009). Mucin-type O-glycans in tears of normal subjects and patients with non-Sjogren’s dry eye. Invest Ophthalmol Vis Sci.

[27] Gooding JR (2015). Adenylosuccinate Is an Insulin Secretagogue Derived from Glucose-Induced Purine Metabolism. Cell Rep.

[28] Grus FH (2005). *SELDI-TOF-MS ProteinChip Array profiling of tea*rs from patients with dry eye. Investigative Ophthalmology & Visual Science.

[29] Yanez-Soto B (2014). Interfacial phenomena and the ocular surface. Ocul Surf.

[30] Addona TA (2009). Multi-site assessment of the precision and reproducibility of multiple reaction monitoring-based measurements of proteins in plasma. Nat Biotechnol.

[31] Kusebauch U (2016). Human SRMAtlas: A Resource of Targeted Assays to Quantify the Complete Human Proteome. Cell.

[32] Conner GE, Wijkstrom-Frei C, Randell SH, Fernandez VE, Salathe M (2007). The lactoperoxidase system links anion transport to host defense in cystic fibrosis. FEBS Lett.

[33] Quaye IK (2008). Haptoglobin, inflammation and disease. Trans R Soc Trop Med Hyg.

[34] Lambeau G, Gelb MH (2008). Biochemistry and physiology of mammalian secreted phospholipases A2. Annu Rev Biochem.

[35] Beers SA (2002). The antibacterial properties of secreted phospholipases A2: a major physiological role for the group IIA enzyme that depends on the very high pI of the enzyme to allow penetration of the bacterial cell wall. J Biol Chem.

[36] Nevalainen TJ, Graham GG, Scott KF (2008). Antibacterial actions of secreted phospholipases A2. Review. Biochim Biophys Acta.

[37] Aho VV, Nevalainen TJ, Saari KM (2002). Group IIA phospholipase A2 content of tears in patients with keratoconjunctivitis sicca. Graefes Arch Clin Exp Ophthalmol.

[38] Chen D (2009). sPLA2-IIa is an inflammatory mediator when the ocular surface is compromised. Exp Eye Res.

[39] Klein RJ (2005). Complement factor H polymorphism in age-related macular degeneration. Science.

[40] Hageman GS (2005). A common haplotype in the complement regulatory gene factor H (HF1/CFH) predisposes individuals to age-related macular degeneration. Proc Natl Acad Sci USA.

[41] Zaidi TS, Zaidi T, Pier GB (2010). Role of neutrophils, MyD88-mediated neutrophil recruitment, and complement in antibody-mediated defense against Pseudomonas aeruginosa keratitis. Invest Ophthalmol Vis Sci.

[42] Ballow M, Donshik PC, Mendelson L (1985). Complement proteins and C3 anaphylatoxin in the tears of patients with conjunctivitis. J Allergy Clin Immunol.

[43] Tsubota K (2017). New Perspectives on Dry Eye Definition and Diagnosis: A Consensus Report by the Asia Dry Eye Society. Ocul Surf.

[44] Ji YW (2017). Lacrimal gland-derived IL-22 regulates IL-17-mediated ocular mucosal inflammation. Mucosal Immunol.

[45] Cox J (2011). Andromeda: a peptide search engine integrated into the MaxQuant environment. J Proteome Res.

[46] Nelissen H (2015). Dynamic Changes in ANGUSTIFOLIA3 Complex Composition Reveal a Growth Regulatory Mechanism in the Maize Leaf. Plant Cell.

[47] Cox J (2014). Accurate proteome-wide label-free quantification by delayed normalization and maximal peptide ratio extraction, termed MaxLFQ. Mol Cell Proteomics.

[48] Deeb SJ, D’Souza RC, Cox J, Schmidt-Supprian M, Mann M (2012). Super-SILAC allows classification of diffuse large B-cell lymphoma subtypes by their protein expression profiles. Mol Cell Proteomics.

[49] Huang da W, Sherman BT, Lempicki RA (2009). Systematic and integrative analysis of large gene lists using DAVID bioinformatics resources. Nat Protoc.

[50] Franceschini A (2013). STRINGv9.1: protein-protein interaction networks, with increased coverage and integration. Nucleic Acids Res.

[51] Ippoliti PJ (2016). Automated Microchromatography Enables Multiplexing of Immunoaffinity Enrichment of Peptides to Greater than 150 for Targeted MS-Based Assays. Anal Chem.

[52] MacLean B (2010). Skyline: an open source document editor for creating and analyzing targeted proteomics experiments. Bioinformatics.

