# Supplementary material for: Proteomic analysis of human lacrimal and tear fluid in dry eye disease
Source: Sci Rep. 2017 Oct 17;7:13363. doi: 10.1038/s41598-017-13817-y (PMC5645331; doi:10.1038/s41598-017-13817-y)
Supplement: Supplementary file 1 — Supplementary Information [file 41598_2017_13817_MOESM1_ESM.pdf]

SUPPLEMENTARY INFORMATION for

Proteomic analysis of human lacrimal and tear fluid in dry eye disease

Jae Hun Jung[1], Yong Woo Ji[2], Ho Sik Hwang[3], Jae Won Oh[1], Hyun Chang Kim[4], Hyung Keun Lee[2]*, Kwang Pyo Kim[1]*

[1]Department of Applied Chemistry, College of Applied Science, Kyung Hee University, Yongin, Korea
[2]Institute of Vision Research, Department of Ophthalmology, Yonsei University College of Medicine, Seoul, Korea
[3]Department of Ophthalmology, Chuncheon Sacred Heart Hospital, Hallym University, Chuncheon, Korea
[4]Department of Preventive Medicine, Yonsei University College of Medicine, Seoul, Korea

*Corresponding authors

Corresponding to:
Kwang Pyo Kim, Ph.D.,
Department of Applied Chemistry, Kyung Hee University, Yongin, Gyeonggi-do 463-707, Korea
Phone: 82-31-201-2419, Fax: 82-31-201-2340
E-mail: kimkp@khu.ac.kr

Hyung Keun Lee, M.D.,
Department of Ophthalmology, Gangnam Severance Hospital, Yonsei University College of Medicine, 211 Eonju-ro, Gangnam-gu, Seoul, Korea 135-720
Phone: 82-2-2019-3444, Fax: 82-2-3463-1049
E-mail: shadik@yuhs.ac

SUPPLEMENTARY FIGURES

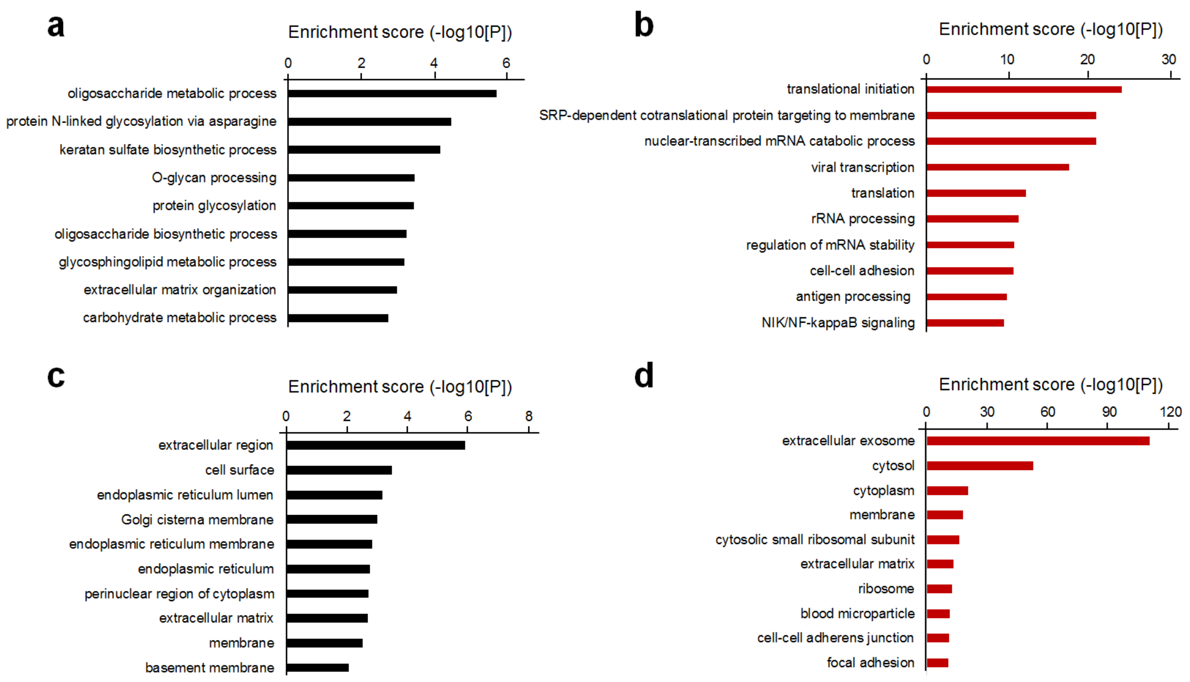

Supplementary Fig S1. Enriched biological processes of **(a)** uniquely identified proteins in TF (# of 316) and **(b)** LF (# of 599) and cellular component of **(c)** uniquely identified proteins in TF and **(d)** LF.

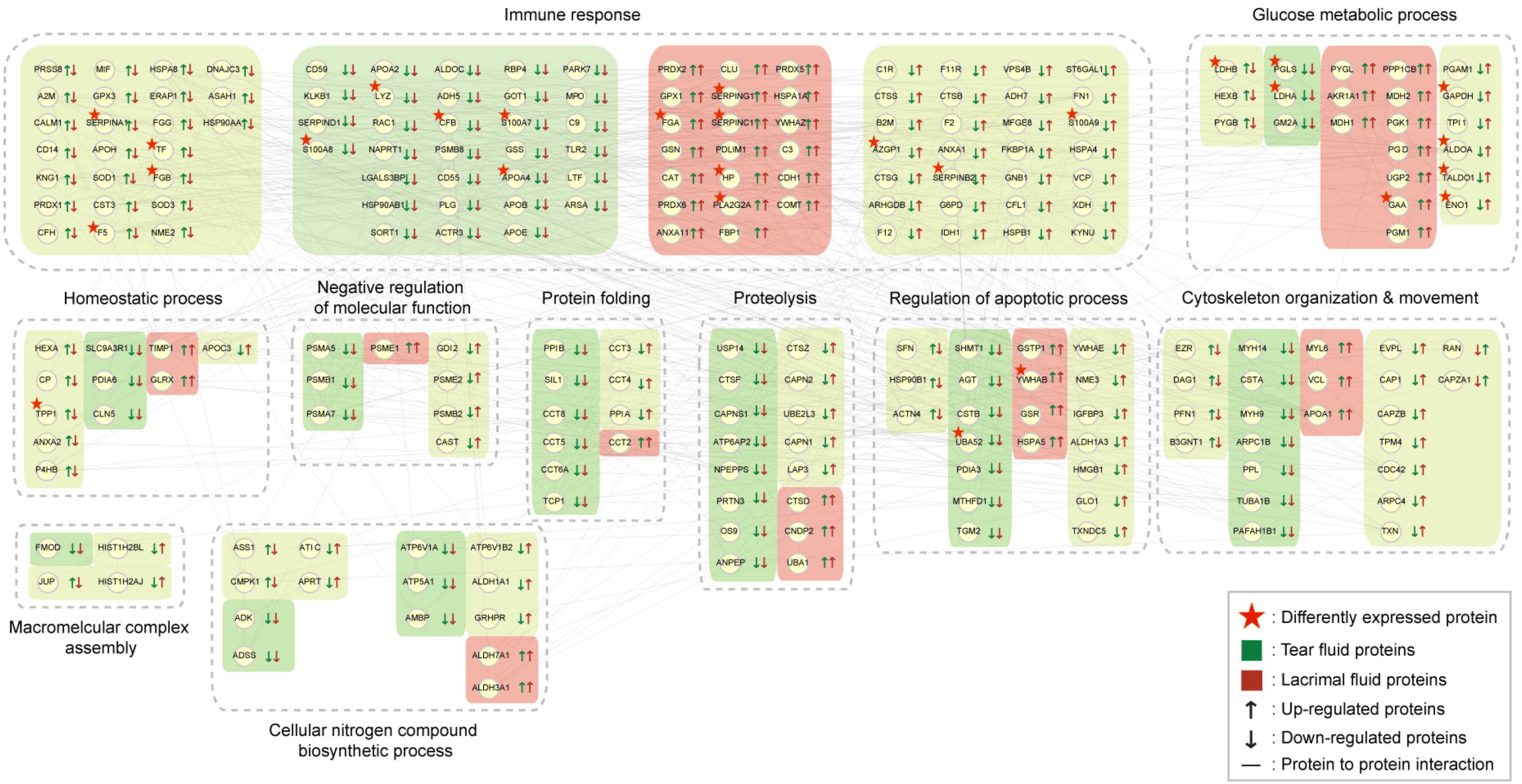

Supplementary Fig S2. Network showing protein-protein interactions and alteration patterns in proteins commonly identified in LF and TF. The green and red arrows represent the changes in TF and LF, respectively. Commonly increased proteins are in the red box, commonly reduced proteins are in the green box, and conversely expressed proteins are in the green box. Proteins were grouped by each biological process. The star marks a protein classified as DEP in TF or LF.

Table legend

Table 1. Clinical parameters for classification of dry eye disease and control patients at each proteomic analysis.

Table 2. Differentially expressed marker proteins obtained from MRM assay consistent with DEPs in LFQ data (** Fold-change > 1.5, p value < 0.05, AUC > 0.7)*.

Supplementary Table S1.

In total, 1764 proteins were identified in the TF and LF. For each protein, the Uniprot accession number and gene name are shown.

Supplementary Table S2.

In total, 142 TF proteins and 137 LF proteins showing altered protein abundances in DED cases compared to in controls. Gene symbols, Log2 fold-change and descriptions of the proteins are provided.

Supplementary Table S3.

Comparison of changes in protein abundance with previous studies. (a) Proteins reported to be differentially expressed in DED cases from four previous tear proteomic studies were compared with our data. 'UP' and 'DOWN' represent a significant difference (>1.5-fold-change in abundance) between DED and controls. (b) Thirty-seven proteins showing consistent protein expression changes in DED.

Supplementary Table S4.

GO-BPs represented by the up-regulated proteins in (a) TF and (b) LF. KEGG pathways enriched by up-regulated proteins in (c) TF and (d) LF. Ivory shade in each cell indicates representative process obtained from 'REVIGO', while cells below ivory shaded rows indicate subprocesses of

the representative processes. ‘Count’ represents the number of proteins associated with the corresponding GO-BPs and KEGG pathways. The given *p*-values imply the significance of being enriched by the up-regulated proteins. ‘Genes’ show the list of genes involved in the GO-BPs and KEGG pathways.

Supplementary Table S5.

GO-BPs represented by the down-regulated proteins in (a) TF and (b) LF. KEGG pathways enriched by down-regulated proteins in (c) TF. Ivory shade in each cell indicates representative process obtained from 'REVIGO', while cells below ivory shaded rows indicate subprocesses of the representative processes. ‘Count’ represents the number of proteins associated with the corresponding GO-BPs and KEGG pathways. The given *p*-values indicate the significance of being enriched by the up-regulated proteins. 'Genes' shows the list of genes involved in GO-BPs and KEGG pathways.

Supplementary Table S6.

Dynamic MRM transition lists of target proteins. Precursor ion (Q1) and product ion (Q3) including collision energy and retention time information are listed.

Supplementary Table S7.

Fold-change between DED and CT in (a) TF and (b) LF. The yellow shades represent DEPs in MRM assay (Fold-change > 1.5 and *p*-value < 0.05) which are consistent with the LFQ data.
